# Polyether ionophore resistance in a one health perspective

**DOI:** 10.3389/fmicb.2024.1347490

**Published:** 2024-01-29

**Authors:** Rikki Franklin Frederiksen, Jannice Schau Slettemeås, Silje Granstad, Karin Lagesen, Mariel G. Pikkemaat, Anne Margrete Urdahl, Roger Simm

**Affiliations:** ^1^Department of Animal Health, Welfare and Food Safety, Norwegian Veterinary Institute, Ås, Norway; ^2^Wageningen Food Safety Research, Wageningen University and Research, Wageningen, Netherlands; ^3^Department of Biosciences, University of Oslo, Oslo, Norway

**Keywords:** antimicrobial resistance, polyether ionophore, one health, vancomycin resistance, anticoccidials, coccidiostats, growth promoters

## Abstract

Antimicrobial resistance is a major threat to human health and must be approached from a One Health perspective. Use of antimicrobials in animal husbandry can lead to dissemination and persistence of resistance in human pathogens. Polyether ionophores (PIs) have antimicrobial activities and are among the most extensively used feed additives for major production animals. Recent discoveries of genetically encoded PI resistance mechanisms and co-localization of resistance mechanisms against PIs and antimicrobials used in human medicine on transferrable plasmids, have raised concerns that use of PIs as feed additives bear potential risks for human health. This review summarizes the current knowledge on PI resistance and discusses the potential consequences of PI-usage as feed additives in a One Health perspective.

## 1 Introduction

Antimicrobial resistance (AMR) poses a serious threat to human health. It has been estimated that 1.27 million deaths were caused by bacterial AMR in 2019 (Murray et al., 2022) and the negative effect on human welfare is predicted to escalate in the next decades. Although the main focus on AMR has been on health care settings, it is recognized that veterinary medicine, plant- and animal production, and environmental sectors play an important role in the origin, persistence, and spread of AMR.

Polyether ionophores (PIs) have been used as feed additives for production animals since the early 1970 (Chapman et al., 2010). PIs possess both antibacterial and antiprotozoal activity and are currently used in poultry production worldwide to control severe diseases such as coccidiosis caused by *Eimeria* spp. and necrotic enteritis caused by *Clostridium perfringens* (Martins et al., 2022). The antibacterial activity of PIs has also proven useful to improve feed conversion in ruminants (Callaway et al., 2003; Kim et al., 2014; Scharen et al., 2017). PIs are not used in human medicine due to their cytotoxicity. However, PIs and PI-derivatives with low toxicity are considered for therapeutic treatment of cancer (Huczyński, 2012; Kaushik et al., 2018; Wang et al., 2021) and infectious diseases caused by bacteria (Wollesen et al., 2023), fungi, protozoa, and even virus (Huczyński, 2012; Lin et al., 2021).

Sales and use of PIs are not systematically reported in most countries, making it difficult to estimate the global consumption level (Hansen et al., 2009; Mulchandani et al., 2023). However, based on available data from countries in Europe, North America, and Australia, PIs are among the most extensively used antimicrobial feed additives for production animals across the world (DANMAP 2015, 2016; PHAC, 2016; SWEDRES/SVARM 2016, 2017; FDA, 2023a).

Polyether ionophores are produced and secreted by bacteria of the class Actinomycetia. They are highly lipophilic compounds that form lipid soluble complexes with cations and facilitate their diffusion through biological membranes. This disrupts chemical gradients across membranes and interferes with essential biological processes. The PIs display different ion selectivity for cations abundant in biological systems depending on their structure. Most commonly used PIs in animal husbandry can bind both K^+^ and Na^+^ under artificial conditions, and with the exception of monensin, these PIs prefer K^+^ over Na^+^. Lasalocid, however, has been shown to form complexes with both mono- and divalent cations (Table 1).

**Table 1:** Polyether ionophores commonly used in animal husbandry and their ion selectivity in artificial systems.

Ionophore	Concentration in feed	Producer organism	Mr	Selectivity sequence	References
Narasin	60–70 mg/kg^†^	*Kitasatospora aureofaciens*	765	K^+^ > Na^+^	Caughey et al. (1986)
Salinomycin	50–70 mg/kg^†^	*Streptomyces albus*	751	K^+^ > Na^+^	Rokitskaya et al. (2023)
Lasalocid (X-537A)	75–125 mg/kg^†^	*Streptomyces lasalocidi*	591	K^+^ > Na^+^ > Ca^2+^ > Mg^2+^	Pressman (1968); Antonenko and Yaguzhinsky (1988)
Maduramicin (X-14868A)	5–6 mg/kg^†^	*Actinomadura yumanensis*	934	K^+^ > Na^+^	Liu et al. (1983)
Monensin A	100–125 mg/kg^†^	*Streptomyces cinnamonensis*	671	Na^+^ > K^+^	Pressman (1968); Antonenko and Yaguzhinsky (1988)
Laidlomycin	~110 mg/kg^#^	*Streptovertilicillium olivoreticuli*	698	K^+^ > Na^+^ > Ca^2+^	Gräfe et al. (1989)

^†^Minimum and maximum concentrations of active substance/kg in complete feed for broiler production in the EU (EUR-lex, 2023). ^#^Recommended concentration of active substance/kg for improved feed efficiency in cattle in the United States (FDA, 2023b).

**Figure 1:**
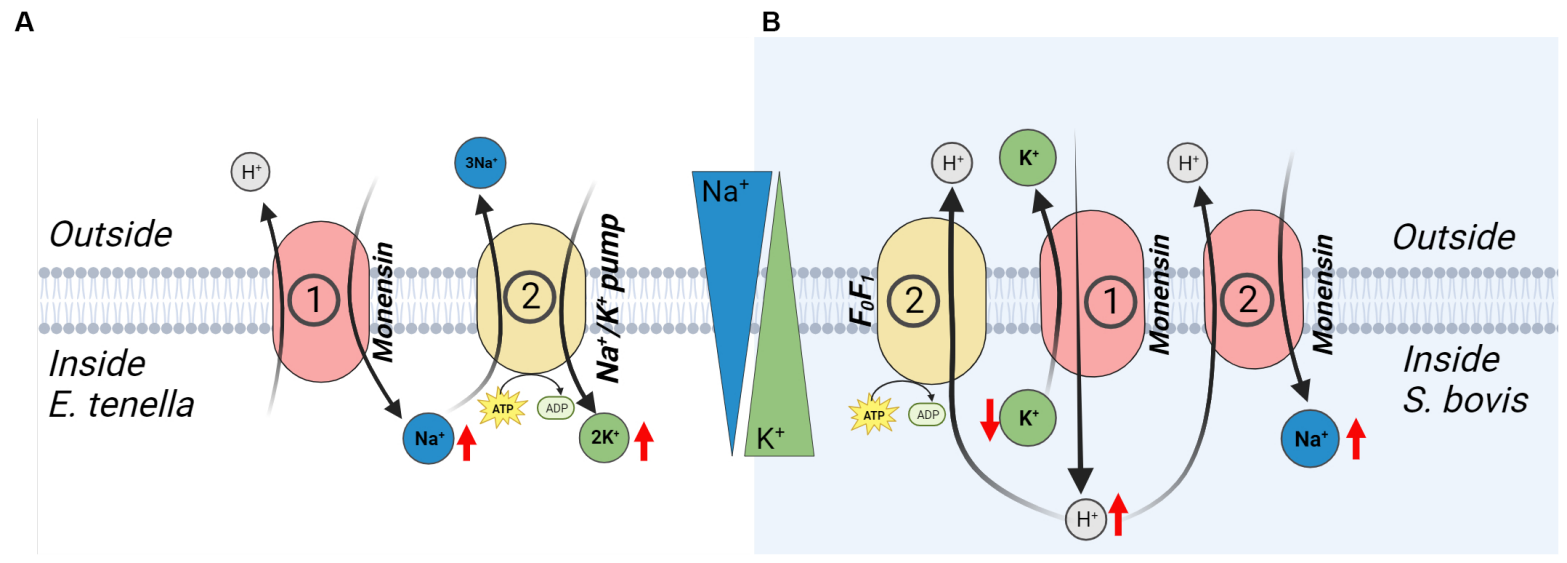
Proposed effects of monensin-mediated ion gradient disruption in *Eimeria tenella* (Smith and Galloway, 1983) parasites **(A)** and in gram-positive *Streptococcus bovis* (Russell, 1987) bacteria **(B)**. Encircled numbers represent suggested sequence of ion transport events. Red arrows indicate direction of change in cystosolic ion concentrations. Cation gradients across a bilayer is displayed by triangles.

The antimicrobial mechanism(s) of these PIs are not fully understood. Independent of the cation preference in artificial systems, it appears that the effect of most PIs used in animal husbandry is disruption of the Na^+^/K^+^ homeostasis and a concomitant change in cytosolic pH (Russell and Strobel, 1989). This could theoretically be a direct effect of the PIs ability to bind both Na^+^ and K^+^ and thereby facilitate transport of Na^+^ into the cell and K^+^ out of the cell, and that the change in pH results from induction of endogenous membrane transport systems to restore the chemical gradients of Na^+^ and K^+^. However, the general opinion is that PIs transport a cation (Na^+^/K^+^) in one direction and H^+^ in the opposite direction. The primary effect would therefore be an intracellular change in Na^+^ or K^+^ gradient and a simultaneous change in pH. It has been hypothesized that the resulting disruption of membrane cation gradients induces compensatory mechanisms to restore cation-homeostasis, including Na^+^/K^+^ ATPase and F-ATPase. The activity of these compensatory mechanisms results in a secondary effect of disrupted Na^+^, K^+^, and H^+^ homeostasis, increased demand for ATP and subsequent tertiary effects on essential cellular processes (Figures 1A,B; Smith and Galloway, 1983; Russell and Strobel, 1989).

It is likely that the PI-mediated disruption of cation gradients directly or indirectly inhibits both primary and secondary membrane transport proteins resulting in disrupted import and export of nutrients, metabolites and xenobiotics. In addition, the disturbances in the intracellular cation concentration may inhibit enzymes involved in essential cellular processes. Although it has been suggested that bacterial growth inhibition could be caused either by energy depletion due to increased demand for ATP or by cell acidification as a result of influx of H^+^ (Russell, 1987), other mechanisms may also be involved.

Polyether ionophores affect bacterial metabolism of carbohydrates, amino acids, and fatty acids. The specific effect of disrupting the chemical gradients across the cytoplasmic membrane varies depending on the cation-dependent processes and the metabolic requirements of the organism. Chow et al. showed that monensin and lasalocid inhibited growth of the gram-negative bacterium *Fibrobacter succinogenes.* They showed that the ATP synthesis was decreased, most likely as an effect of an inability to take up glucose via Na^+^/glucose symporters (Chow and Russell, 1992). PIs have also been shown to reduce amino acid transport in ruminal bacteria (Chen and Russell, 1989; Russell and Strobel, 1989; Van Kessel and Russell, 1992). In contrast, while monensin exposure caused cessation of growth of *Streptococcus bovis in vitro* (Russell, 1987), glucose transport was not inhibited and glucose fermentation continued resulting in continuous ATP production (Russell, 1987). The hypothesized explanation to this observation was that *S. bovis* can use the phosphotransferase system as well as facilitated diffusion for glucose uptake (Russell et al., 1990).

Similar biological effects have been observed in the protozoan parasite *Eimeria tenella* where monensin caused an increase in intracellular concentrations of both Na^+^ and K^+^ (Smith and Galloway, 1983). As specific inhibition of the Na^+^/K^+^-pump increased the K^+^ level, it was proposed that monensin caused an initial uptake of Na^+^ followed by an exchange of intracellular Na^+^ for extracellular K^+^ (Figure 1B). Several anticoccidial modes of PIs have been suggested, such as energy depletion, mitochondrial stress, and inhibition of invasion of enterocytes. However, parasite swelling, and eventual bursting, have been suggested as the most likely mode of action (Smith et al., 1981; Chapman et al., 2010). Whether the osmotic stress survival of bacteria is influenced by PIs has to our knowledge not been shown experimentally.

The specific activity of PIs is influenced by the extracellular conditions. Extracellular cation concentrations promoting the natural electrochemical gradients across the cytoplasm (high [Na^+^], [H^+^], [Ca^2+^]) enhances the activity of ionophores, whereas extracellular cation concentrations equilibrating the intracellular levels (high [K^+^]) decrease the ionophore-activities (Dawson and Boling, 1987; Russell et al., 1988; Chow and Russell, 1992; Van Kessel and Russell, 1992; Wollesen et al., 2023). Considering that the cation concentrations and pH varies along the length of the gastrointestinal tract of animals, it is likely that the antimicrobial effect of PIs differs in different parts of the gastrointestinal tract, and thereby exert different selection pressures on the local microbiota.

## 2 PI-resistance mechanisms in bacteria

Antimicrobial resistance is the ability of bacteria to survive and grow in the presence of antimicrobials (Balaban et al., 2019). Since polyether ionophores have not been used in human medicine, clinical cut-off values have not been established. When we discuss resistance to polyether ionophores in this review, we refer to survival and growth of: (1) a particular strain of bacteria at PI concentrations to which it was previously susceptible, (2) a species at concentrations above the epidemiological cut-off values for that species, or (3) a species at the highest concentration of PI tested in susceptibility assays *in vitro* (intrinsic resistance).

Resistance mechanisms in bacteria are attributed to either intrinsic resistance, where all individuals of a certain type of bacteria can survive and grow in the presence of a specific antimicrobial, or acquired resistance that can arise in a previously susceptible population due to mutations or horizontal transfer of resistance genes. In addition, bacteria can survive high concentrations of antimicrobials due to the formation of persister cells or biofilms. Persister cells are subpopulations of a species with a different physiology (metabolically quiescent) compared to the general bacterial population. Biofilms are bacterial communities embedded in an extracellular matrix consisting of physiologically diverging subpopulations of bacteria (Olivares et al., 2013).

Although bacterial resistance to PIs was described 30 years ago, the mechanism(s) of resistance are poorly understood. Table 2 summarizes the currently known putative and confirmed bacterial resistance mechanisms.

**Table 2:** Putative bacterial polyether ionophore-resistance mechanisms.

Resistance	Bacteria	Target PI	Putative mechanism	References
Intrinsic	Many gram (−)	All PIs	Reduced permeability due to an outer membrane with negatively charged LPS and porins	Nagaraja (1995)
Acquired	*Enterococcus faecium* (+)	Narasin, Salinomycin, and Maduramicin	Ionophore efflux by ABC type transporter encoded by *narAB*	Naemi et al. (2020)
	*Enterococcus faecalis* (+)			
	*Streptomyces lividans* (+)	Tetronasin^§^	Ionophore efflux by ABC type transporter encoded by *tnrB2/B3*	Linton et al. (1994)
	*Staphylococcus aureus* (+)	Nanchangmycin^§^	Regulation of potassium homeostasis by potassium importer encoded by *trkH*^†^, Regulation of membrane integrity by proteins encoded by *sarV*^†^, *mspA*^†^	Wollesen et al. (2023)
	*S. aureus* (+)	Nanchangmycin^§^, Lasalocid, and Salinomycin	Altered metabolism due to mutations in genes *aroC*^#^, *hemB*^#^, *qoxABC*^#^, *ndh2*^#^, and *cyoE*^#^	Wollesen et al. (2023)
	*S. aureus* (+)	Monensin	Altered nucleotide metabolism due to mutations in *apt*^†^, *purR*^†^, and *R*egulation of Na^+^/H^+^ homeostasis by Na^+^/H^+^ antiporter encoded by *mnh*^†^	Dan I. Andersson (personal communication, March 9, 2023; published with permission)
	*Mycolicibacterium aurum* (AF)	Nigericin^§^	Altered gene regulation due to mutations in transcriptional regulator *tetR*^†^, Regulation of Na^+^/H^+^ homeostasis by Na^+^/H^+^ antiporter encoded by *nhaA*^†^	Huang et al. (2017)
	*M. aurum* (AF)	Calcimycin^§^	Altered gene regulation due to mutations in transcriptional regulator *tetR*^†^	Huang et al. (2017)
Altered physiology	*Prevotella bryantii* (−)	Monensin	Cell wall thickening	Callaway and Russell (1999); Rychlik and Russell (2002); Simjee et al. (2012)
	*E. faecium* (+)			
	*E. faecalis* (+)			
	*Clostridium aminophilum* (+)			
	*S. aureus* (+)	Salinomycin, Narasin, Nanchangmycin^§^, and Calcimycin§	Biofilm and persisters	Wollesen et al. (2023)

^†^Mutants isolated under laboratory conditions; ^#^transposon insertion mutant, (−) gram-negative, (+) gram-positive, (AF) acid fast; ^§^ionophores presented to provide insight into potential resistance mechanisms, but not applied in animal production.

### 2.1 PI-resistance in gram-negative bacteria

Gram-negative bacteria generally display intrinsic resistance to PIs due to the nature of their cell envelope. The outer membrane of gram-negative bacteria is impenetrable to many macromolecules and allows passage of solutes through porins. Porins are hydrophilic channels embedded in the outer membrane with a size exclusion limit of approximately 600 daltons. Ionophores are highly lipophilic and in general larger than 600 daltons, making them unable to pass through the porins and the negatively charged LPS of the outer membrane (Nagaraja, 1995).

Intrinsic resistance to PIs is not universal to all gram-negative bacteria. Certain strains of *Bacteroides*, *Fibrobacter*, and *Prevotella* belonging to the ruminal microbiota were sensitive to monensin when grown *in vitro* (Chen and Wolin, 1979; Newbold et al., 1993; Callaway and Russell, 1999, 2000). Some sensitive strains developed resistance after exposure to sub-lethal monensin concentrations through unknown mechanisms, while others remained sensitive (Callaway and Russell, 1999, 2000). *Prevotella ruminicola* grown in the presence of increasing concentrations of tetronasin developed resistance to tetronasin, lasalocid, and monensin and to a lesser extent to the glycopeptide avoparcin. The resistant mutants did not lose the resistance phenotype after subculturing in the absence of ionophores and bound less radioactively labeled ionophore. Reduced metabolism of tetraphenylalanine (Mr = 607), but unaffected metabolism of triphenylalanine (Mr = 460), indicated reduced penetration through the outer membrane, and reduced porin exclusion limit was suggested as the mechanism of resistance (Newbold and Wallace, 1989).

### 2.2 PI-resistance in gram-positive bacteria and mycobacteria

In contrast to gram-negative bacteria, gram-positive bacteria do not possess a protective outer membrane. Although the outer peptidoglycan layer of gram-positive bacteria can be relatively thick, it is porous and permits diffusion of small molecules, and this allows for the lipophilic ionophores to readily dissolve into the cell membrane of gram-positive bacteria (Rutkowski and Brzezinski, 2013).

#### 2.2.1 Plasmid mediated PI-resistance mechanisms

Although the biological role of PI production is not yet established (Bakker, 1979; Kevin et al., 2009), it is likely that the antibacterial activity of ionophores improves the competitiveness of PI secreting bacteria in their habitats. The secretion of ionophores into the environment suggests that PI producing bacteria concomitantly express protective mechanisms of self-resistance. A self-resistance mechanism against the PI tetronasin was identified in 1994 by screening of a genomic library from the tetronasin-resistant *Strepmomyces longisporoflavus* in the tetronasin-susceptible species *Streptomyces lividans* (Linton et al., 1994). Tetronasin-resistance was associated with a DNA region containing the *tnrB2/B3* operon encoding an ATP-binding cassette (ABC) transporter consisting of ATPase (TnrB2) and permease (TnrB3) subunits. Later, the presence of plasmid-encoded homologs of *tnrB2/B3* were identified in *Enterococcus faecium* isolates from Swedish and Norwegian poultry, and the presence of these genes on large mobile plasmids correlated with resistance to narasin (Nilsson et al., 2016). Naemi et al. (2020) cloned the putative narasin resistance genes, coined *narAB*, into a cloning vector under control of its natural promoter and unequivocally showed that this operon was sufficient to confer resistance to the PIs narasin, salinomycin and maduramicin, but not to monensin. Interestingly, Naemi et al. also showed that the *narAB* operon was transcriptionally upregulated by exposure to narasin, which potentially reduces the fitness cost associated with carrying the operon in PI-free conditions.

In *E. faecium* isolated from broilers in Norway (Sletvold et al., 2007, 2008, 2010), Sweden (Nilsson et al., 2016), Denmark (Leinweber et al., 2018), and the Netherlands (Pikkemaat et al., 2022), the *narAB* operon was located on plasmids belonging to the broad-host-range inc18-group (Gilmore et al., 2014). Inc18 plasmids are widespread in isolates from the environment, the clinic, and domestic animals (Kohler et al., 2018). They are naturally occurring in streptococci and enterococci, often carry resistance genes, and have been shown to be transferable from enterococci to staphylococci (Kohler et al., 2018). The assembled NarAB-encoding plasmids published to date, vary in size and carry mobile elements such as transposons and insertion sequences. These plasmids share only limited gene synteny. However, the *narAB* operon is often associated with a full or truncated ω-ε-ζ toxin-antitoxin system and flanked by insertion sequences, such as IS1216 (Sletvold et al., 2007, 2008, 2010; Leinweber et al., 2018). Filter mating experiments demonstrated that NarAB encoding plasmids were transferable between *E. faecium* strains despite the lack of apparent plasmid encoded transfer systems (Dahl et al., 2007; Nilsson et al., 2012; Leinweber et al., 2018; Naemi et al., 2020). Leinweber et al. (2018) observed that the NarAB encoding plasmid was transferred by conjugation along with a larger co-residing conjugative plasmid suggesting that the larger plasmid acted as a helper plasmid. In Dutch *E. faecalis* isolates, *narAB* was most often localized on large plasmids of the RepA_N family (Pikkemaat et al., 2022). RepA_N plasmids display a relatively broad distribution but appear to be adapted to their host and display restricted transferability to other species (Weaver et al., 2009).

The resistance mechanism(s) of NarAB and TnrB2/B3 have not yet been characterized. The ABC transporter superfamily is an ancient family of membrane transporters that utilizes the energy released from hydrolysis of ATP to drive transport of substrates against a concentration gradient. ABC-transporters transport a wide range of substrates including ions, nutrients, xenobiotics, and secondary metabolites, including antibiotics. It is obvious to assume that NarAB and other TnrB2/B3 homologs function as drug efflux proteins, but this has not yet been proven experimentally.

The origin of *narAB* is not known. The occurrence of *narAB* in certain subpopulations of enterococci and the general localization of this operon on mobilizable plasmids suggests acquisition by horizontal gene transfer. The similar function of NarAB and TnrB2/B3 suggests that NarAB may have originated as a self-resistance mechanism in PI-producing bacteria. However, the permease subunit NarB (Accession: QHA94815.1) displays 33% identity to TnrB3 (Accession: CAA52013.1) of *Strepmomyces longisporoflavus* and 30% identity to a putative self-resistance gene (Accession: WP_030553101.1) of the narasin producing *Kitasatospora aureofaciens* (our unpublished data). This suggests that if the gene originates from horizontal gene transfer from PI-producing bacteria it would have been an early event predating the use of ionophores in animal husbandry. Alternatively, *narAB* originates from a so far unidentified bacterium, and it cannot be excluded that it has divergently evolved from an operon that is intrinsic to a species of enterococci.

#### 2.2.2 Mutations giving rise to PI-resistance

In a screen for natural compounds displaying *in vitro* anti-mycobacterial activity, Huang et al. (2017) identified the PIs nigericin, calcimycin (A23187), and salinomycin as hits. Spontaneous mutants of *Mycobacterium* spp. resistant to nigericin and calcimycin were isolated on selective plates. Deleterious mutations in a *tetR* family regulator resulted in resistance to both PIs, while a non-synonymous mutation in *nhaA* resulted in resistance to nigericin. Mutation of the *tetR* regulator led to increased transcription of an RND family efflux pump hypothesized to export the PIs, while mutation in *nhaA* encoding a homolog of Na^+^/H^+^-antiporter likely compensates for a disrupted sodium gradient.

Wollesen et al. (2023) grew *Staphylococcus aureus* in the presence of the PI nanchangmycin with the intention to isolate mutants resistant to PIs and to identify potential resistance mechanisms. Nanchangmycin-resistant mutants carried mutations in genes encoding a potassium importer TrkH, a transcriptional activator involved in regulating autolysis SarV, and a membrane stabilizing protein MspA. Interestingly, the authors did not detect cross-resistance in these mutants to salinomycin, lasalocid, or calcimycin. To gain further insight into the resistance mechanisms, a methicillin-resistant *S. aureus* transposon mutant library was screened for increased sensitivity to lasalocid, salinomycin, calcimycin, and nanchangmycin. Mutations in the electron transport chain (ETC) genes *qoxABC*, *nhd2*, and *cyoE*, conferred a modest increase in sensitivity toward lasalocid, salinomycin, and nanchangmycin (Wollesen et al., 2023). These findings indicate a role of the ETC in PI-resistance in *S. aureus*, potentially to meet the high demand for energy to uphold cation homeostasis across the cell membrane.

Recently, spontaneous mutants with reduced susceptibility to monensin were isolated after growth of clinical *S. aureus* isolates under laboratory conditions. Mutations were found in different genes, such as *apt* encoding an adenine phosphoribosyltransferase, *purR*, encoding a repressor of nucleotide biosynthesis, and non-synonymous mutations in different genes of the *mnh* operon encoding a Na^+^/H^+^ antiporter (Dan I. Andersson, personal communication, March 9, 2023; published with permission). While *apt* and *purR* theoretically are involved in compensating for increased need for ATP to counteract a PI-induced cation imbalance, the *mnh* mutations are likely directly compensating for a disrupted transmembrane chemical gradient.

### 2.3 PI-resistance due to altered bacterial physiology

Persister cells and biofilm formation are mechanism considered to play key-roles in persistence of bacteria in different environments including the human host (chronic infections) and causing antimicrobial treatment failures (Olivares et al., 2013). Wollesen et al. (2023) analyzed the antimicrobial effect of PIs on a laboratory-induced persister phenotype and preformed biofilms of *S. aureus.* They observed that in general both persister cells and biofilms were more resistant to PIs compared to exponentially growing bacteria. Interestingly, persister cells were susceptible to lasalocid, and biofilms were susceptible to lasalocid, calcimycin, and nanchangmycin (Wollesen et al., 2023).

In the gram-positive bacteria *Clostridium aminophilum F*, *Clostridium perfringens*, *E. faecalis*, and *E. faecium* and in the gram-negative species *Prevotella ruminants*, adaptation to growth in monensin was associated with thickening of the cell wall (Callaway and Russell, 1999; Rychlik and Russell, 2002; Simjee et al., 2012). The observed increase in cell wall thickness was reversed after passage in monensin-free medium and the authors concluded that monensin resistance was due to physiological changes rather than mutations (Simjee et al., 2012). However, it should be noted that this was not confirmed by whole genome sequencing.

## 3 Relationship between PI resistance and resistance to medically important antimicrobials

Since PIs are currently not utilized to combat bacterial infections in humans due to cytotoxicity, the prevalence of PI-resistance in bacterial isolates from animals has not been considered a threat to public health. However, use of PIs in animal husbandry and PI-resistance can contribute to resistance to medically important antimicrobials if PI-resistance confers cross-resistance or co-resistance to antimicrobials used to treat infections in humans. While cross-resistance occurs in a microbe carrying a resistance mechanism conferring resistance to structurally different antibiotics, co-resistance arises if genes encoding resistance mechanisms for different antimicrobials are genetically linked.

### 3.1 Sparse evidence of cross-resistance between PIs and medically important antimicrobials

The ruminal bacterium *C. aminophilum* that had been adapted to grow in monensin or lasalocid, was only resistant to the cell-wall acting bacitracin out of 16 tested medically relevant antibiotics (Houlihan and Russell, 2003). Interestingly, these results are in agreement with a strong association between narasin resistance and bacitracin resistance as discovered in *Enterococcus* spp. isolates from broilers in Norway (NORM/NORM-VET 2004, 2005). However, it was shown that NarAB does not confer cross-resistance to bacitracin, suggesting two separate AMR mechanisms for these antimicrobials in enterococci (Naemi et al., 2020). As described above, the gram-negative bacterium *P. ruminicola*, adapted to grow in the presence of tetronasin, displayed a moderately reduced (65%) susceptibility to the glycopeptide avoparcin (Newbold et al., 1992). The cross-resistance between tetronasin and avoparcin was likely due to changes in the porins of the outer membrane. Some of the mutants of *S. aureus* that are resistant to monensin due to mutations in *apt*, *purR*, or *mnh* displayed minor changes in resistance to antimicrobials used to treat infections in humans (Dan I. Andersson, personal communication, March 9, 2023; published with permission). These resistance mechanisms have not been characterized in detail and a mechanism of cross-resistance to medically relevant antimicrobials have not been confirmed.

Naemi et al. (2020) tested the resistance profile of *E. faecium* carrying the *narAB* operon on a plasmid under control of the natural promoter and compared it to the resistance profile of the isogenic strain carrying the vector control. They detected no difference between the two strains in minimum inhibitory concentration (MIC) values of any of the tested medically important antimicrobials, suggesting that NarAB does not confer resistance to antimicrobials used in human medicine (Naemi et al., 2020).

### 3.2 Co-resistance of PIs and medically important antimicrobials

Vancomycin-resistant *E. faecium* has regularly been isolated at low levels from broilers in Norway and Sweden using a selective isolation method (NORM/NORM-VET 2004, 2005; SWEDRES/SVARM 2013, 2014). All of these vancomycin-resistant enterococci (VRE), which carry *vanA*, were also resistant to narasin and carried the *narAB* genes (Nilsson et al., 2012; Simm et al., 2019). Filter mating experiments showed that vancomycin and narasin resistance were frequently co-transferred to a recipient (Nilsson et al., 2016; Naemi et al., 2020). Comparative genomics revealed that *narAB* and *vanA* can be physically linked on transferrable plasmids (Johnsen et al., 2005; Nilsson et al., 2016).

A study from the Netherlands on 35 *E. faecium* and 61 *Enterococcus faecalis* isolates from poultry products found statistically significant correlations between phenotypic resistance to salinomycin, tetracycline, and erythromycin in both species (Pikkemaat et al., 2022). Sequencing of a selection of 20 isolates revealed that *narAB* was present in all of the salinomycin-resistant isolates of both *E. faecium* and *E. faecalis*. In the *E. faecalis* isolates, *narAB* was physically linked with *ermB* (conferring macrolide resistance) and *tet(L)* and *tet(M)* or *tet(O)* (*tet*-genes confer resistance to tetracycline) on plasmids (Pikkemaat et al., 2022), confirming the co-occurrence of resistance genes. The correlation between salinomycin and tetracycline resistance aligns with a metagenomics study in which narasin-fed chickens were enriched for bacteria encoding tetracycline resistance genes, suggesting that narasin and tetracycline resistance are co-selected (Plata et al., 2022). Taken together these data provide strong evidence for transferrable co-resistance of PIs and medically important antimicrobials.

## 4 Prevalence of PI-resistant bacteria in animals and risks for human health

The worldwide prevalence of PI-resistance is challenging to assess because resistance to PIs is not reported systematically, and large-scale surveys of resistance has not been performed. However, a few countries in Europe have regularly reported on the prevalence of PI-resistance in indicator bacteria such as the opportunistic pathogens *E. faecium* and *E. faecalis* isolated from animals or animal products.

### 4.1 Prevalence of PI-resistant bacteria in animals

**Figure 2:**
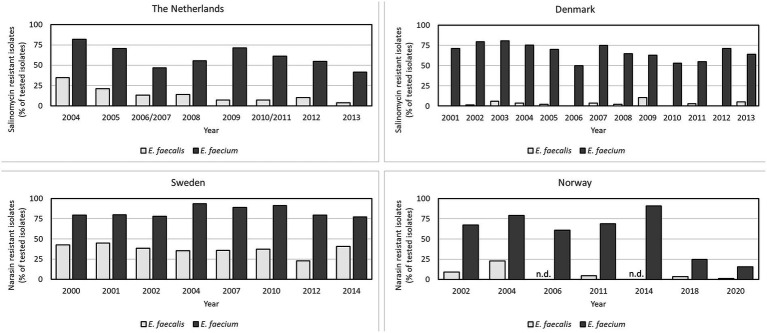
Comparison of the proportion of polyether ionophore resistant isolates from conventionally reared broilers of *Enterococcus faecium* and *Enterococcus faecalis* in four European countries sorted by year. The data were retrieved from the reports of the surveillance programs of each country (MARAN, The Netherlands; DANMAP, Denmark; SVARM, Sweden; NORM-VET, Norway). Cut-off values used were > 4 mg/L for salinomycin, and > 2 mg/L for narasin. Note that these cut-off values are higher than the values proposed to correlate with the *narAB* resistance genes by Pikkemaat et al. (2022) and Nilsson et al. (2016) and the results likely underestimate the true prevalence of PI resistant isolates in broiler populations.

In a Dutch report from 2022, the authors analyzed the antimicrobial susceptibility of *E. faecium* and *E. faecalis* from an in-house collection of isolates gathered in the years 2013, 2016, 2018, and 2020. They reported that 31% of *E. faecium* and 23% of *E. faecalis* isolates collected from broilers and broiler products of conventionally reared poultry displayed a MIC of salinomycin >2 mg/L, while 48.6% of *E. faecium* and 47.5% of *E. faecalis*, displayed a MIC value >1 mg/L. The authors performed whole genome sequencing of a subset of the isolates and discovered that all isolates with a MIC >1 mg/L carried plasmids encoding the PI-resistance genes *narAB* (Pikkemaat et al., 2022). Based on this, it was suggested that the cut-off value for salinomycin defining a non-wild-type should be adjusted to >1 mg/L. The Dutch AMR monitoring program (MARAN) historically used a MIC of 4 mg/L as cut-off for resistance and reported the number of resistant isolates for each year. The proportion of resistant *E. faecium* and *E. faecalis* isolates from Dutch broilers varied between 41.3–81.7% and 3.9–34.5%, for *E. faecium* and *E. faecalis*, respectively, in the years 2004–2014 (Figure 2; MARAN, 2023). Applying a cut-off of >1 mg/L in place of >4 mg/L to the MIC results from the last MARAN report to document salinomycin resistance, increased resistance rates from 5.6 to 61.9% and 38.5 to 84.6% for *E. faecalis* and *E. faecium*, respectively (MARAN, 2014). The Danish surveillance program (DANMAP) reported during the same period that their proportion of isolates from broilers with an MIC of salinomycin >4 mg/L varied between 50 and 74.8% for *E. faecium* and 0–10.5% for *E. faecalis* (Figure 2; DANMAP, 2023). Considering the results of Pikkemaat et al. that cut-off values as low as 1 mg/L correlated with occurrence of the *narAB* resistance operon, the occurrence of PI-resistant enterococci in both Denmark and the Netherlands must be regarded as significantly higher than previously reported.

In Norway and Sweden, narasin was used in the test-panels for surveillance of PI resistance in indicator enterococci. In Sweden, 77% of *E. faecium* and 27% of *E. faecalis* isolated from broilers in 2014 were considered narasin-resistant with MIC over the EUCAST epidemiological cut-off (ECOFF) value (>2 mg/L) (SWEDRES/SVARM 2013, 2014). The prevalence of resistant isolates varied in the years 2000–2014 with 77–93.3% for *E. faecium* and 22.7–44.9% for *E. faecalis* (Figure 2) (SWEDRES-SVARM, 2023). This is similar to the situation in Norway, where 61–91% of the *E. faecium* isolates from broilers were resistant to narasin in the years 2002–2014 (Figure 2) (NORM/NORM-VET, 2023). Since PIs are not currently used in human medicine, most countries have removed PIs from the AMR test panels in monitoring of indicator enterococci. However, due to a decision by the poultry industry in Norway to remove PIs as feed additives in conventional rearing of broilers in 2015, occurrence of narasin resistance in *E. faecium* and *E. faecalis* was monitored in 2018 and 2020 to follow the development after discontinuation. The occurrence of narasin-resistant *E. faecium* isolates was reduced from >90% in 2014 to 24.7% in 2018 and 15.6% in 2020 (NORM/NORM-VET 2018, 2019; Simm et al., 2019; NORM/NORM-VET 2020, 2021). These data strongly suggests that in-feed PIs select for PI-resistant enterococci in broilers, a conclusion that is supported by a controlled study comparing occurrence of PI-resistant enterococci in broilers fed diets with and without PIs (Simm et al., 2019).

DANMAP reported that 0 of 1,349 *E. faecalis* isolates and 1 of 1,217 *E. faecium* isolates collected from pigs between 2004 and 2013 had a MIC of salinomycin >4 mg/L (DANMAP, 2023). The Norwegian monitoring program for AMR in the veterinary sector (NORM-VET) reported that 0 and 3.2% of *E. faecalis* and *E. faecium* pig isolates were resistant to narasin (MIC >2 mg/L) in 2004, 2008, and 2009 (NORM/NORM-VET 2004, 2005; NORM/NORM-VET 2008, 2009; NORM/NORM-VET 2009, 2010). Similarly, narasin resistance was detected in only 2% of *E. faecium* isolates from layers in Norway in 2013 (NORM/NORM-VET 2013, 2014). *Enterococcus faecium* and *E. faecalis* collected from turkeys in Norway in 2007, 2013, and 2020 displayed 76.2 and 7.3% narasin-resistant isolates, respectively (NORM/NORM-VET 2007, 2008; NORM/NORM-VET 2013, 2014; NORM/NORM-VET 2020, 2021). PIs have not been used in rearing of pigs or layers in the sampling period, but turkeys were fed a diet containing monensin. In the Netherlands, the prevalence of salinomycin resistant enterococci has steadily declined since 2006, when use of salinomcyin was banned in rearing of pigs. These data further support that in-feed PIs select for PI-resistant bacteria.

In Norway, the glycopeptide avoparcin was used as a feed additive in broiler production between 1986 and 1995 (Borgen et al., 2000b). Vancomycin resistance provides cross resistance against avoparcin and use of avoparcin as a feed additive avidly selected for VRE in Norwegian broilers (Kruse et al., 1999). In 2000, it was still possible to isolate VRE from 99% of sampled broiler farms in Norway by plating samples directly onto agar supplemented with vancomycin (Borgen et al., 2000a). This selective method has also been used in the Swedish and Norwegian surveillance programs. Data from Norway showed decreasing detectable occurrence of VRE in broilers between 2002 and 2014 and non-detectable levels since 2018. Avoparcin has not been used in Sweden since the ban of antimicrobial growth promoters in 1986. The occurrence of VRE in Swedish broiler flocks first increased in the early 2000, reached a peak at >40% in 2005 (Nilsson et al., 2019) and has decreased since then. VRE were detected in 11% of samples from Swedish broilers in 2015 (Nilsson et al., 2019) and 6% of the samples in 2020 (SWEDRES/SVARM 2020, 2021). Corresponding data does not exist for other European countries since the national surveillance programs have not used the selective method of isolation.

Considering that all VRE in Norway and Sweden are co-resistant to narasin, the fact that VRE has not been detected in Norwegian broilers after the discontinuation of prophylactic use of in-feed narasin, and the continued detection of VRE in Swedish broilers fed narasin in the same time period, is strong evidence that narasin selects for VRE in these broiler production systems. It should be noted that there has been a continuous decline in occurrence of VRE in both Norway (between 2002 and 2014) and Sweden (between 2005 and 2020) despite use of narasin as a feed additive. This does not contradict the selection pressure of narasin for VRE co-resistant to PIs. VRE have only been detected occasionally and at very low levels by non-selective methods (0.14 and 0.75% in Sweden and Norway, respectively from 2000 to 2020). This means that VRE represent a very small proportion of the narasin resistant population of enterococci. Therefore, it is fair to assume that the apparent occurrence of VRE decreases over time even under narasin selection pressure as long as VRE are not re-introduced into the broiler production system.

### 4.2 Occurrence of PI-resistant bacteria in humans and risks for human health

One in 50 *E. faecium* isolates from healthy human volunteers sampled in Denmark in 2005 displayed an MIC of salinomycin >4 mg/L (DANMAP 2005, 2006). Interestingly, one isolate from the same material was also vancomycin resistant. Considering that all VRE isolated from broilers in Norway are also PI-resistant, it is tempting to speculate that the VRE isolate from healthy humans in Denmark was the same isolate that was salinomycin resistant. Furthermore, in depth analysis of WGS data from three large collections of enterococci reveals that *narAB* is also found in human isolates, including clinical isolates, though at low prevalence (Gouliouris et al., 2018; Arredondo-Alonso et al., 2020; Pöntinen et al., 2021). This clearly shows that even though PIs have never been used in human medicine, PI resistant isolates can colonize humans and cause invasive infections.

Several reports have demonstrated the temporary colonization of human intestine with antimicrobial-resistant *E. faecium* and *E. faecalis* transmitted by direct or indirect animal contact or by meat consumption (Bortolaia and Guardabassi, 2015) including narasin-resistant VRE (Johnsen et al., 2005; Sørum et al., 2006). Such temporary colonization could allow for transfer of antimicrobial resistance genes from isolates of animal origin to bacteria in the human host, such as in the case of *vanA*-encoding *E. faecium* (Lester et al., 2006). Plasmids encoding narasin and vancomycin resistance have been shown to transfer readily from poultry derived VRE to human isolates of *E. faecium* in a mouse model (Dahl et al., 2007). Transfer was observed within a day after inoculation of the donor strain. Plasmids from enterococci can also spread to other human pathogens. Experimental transfer of vancomycin resistance from *E. faecium* to *S. aureus* has for instance been demonstrated on mouse skin (Noble et al., 1992). Clinical isolates of vancomycin-resistant *S. aureus* (VRSA) from humans are often accompanied by VRE (Cong et al., 2020) and genomic comparisons have demonstrated that the *vanA* locus can transfer from plasmids of VRE and be stably integrated into the chromosome of *S. aureus* to create VRSA (Haas et al., 2023). This demonstrates that temporary colonization of humans by resistant bacteria of animal origin can pose a threat to human health even if the animal derived strain does not stably colonize the human host or cause infection in humans.

High prevalence of PI-resistance has mostly been documented for the indicator bacteria and prevalent nosocomial opportunistic pathogens, *E. faecium* and *E. faecalis*, collected from farm animals in Scandinavia and the Netherlands. However, PI resistance is likely globally widespread in enterococci from animals fed diets supplemented with PIs, and potentially also present in other opportunistic pathogens, such as *S. aureus*. So, although the available evidence does not indicate a major transmission of PI resistant bacteria from poultry to humans, transmission does occur and may be more pronounced in countries with practices for animal husbandry that differ from the regulations set by the European Union.

There are still major knowledge gaps on the risks associated with PI resistance for human health: (1) The global consumption level of PIs and hence the potential selection pressure for PI-resistance in different parts of the world are unknown since data on use of PIs in animal husbandry is not reported in most countries; (2) The frequency of PI resistance among isolates from human infections is unknown since these isolates are rarely tested for PI susceptibility; (3) The carriage rate of PI-resistant bacteria in humans is unknown. The proportion of PI-resistant bacteria in a microbiota not exposed to a selection pressure may be low and selective identification methods are required to assess the prevalence of PI resistance; (4) The distribution of PI resistance and the potential for dissemination of the resistance mechanism(s) among human pathogens are unknown. PI-resistance has mainly been tested in gram-positive indicator bacteria (enterococci) from production animals. Systematic studies from different environments and different parts of the world are needed to properly assess the risks; (5) The PI resistance mechanism(s) must be identified and characterized in detail and the full complement of resistance mechanisms to medically important antimicrobials that exist on PI-resistance plasmids should be determined.

## 5 Conclusion

Bacteria can become resistant to PIs through horizontal gene transfer of resistance genes and by mutations of intrinsic genes. So far, none of the putative resistance mechanisms have been characterized in detail. However, accumulating evidence suggests that several mechanisms can confer resistance to PIs, including efflux of PIs from the cell, upregulation of cation transporters counteracting the action of the PI and reduced permeability of PIs into the cell. The *narAB* operon is localized on transferrable plasmids in the human opportunistic pathogens *E. faecium* and *E. faecalis*. These plasmids have been shown to carry various resistance mechanisms to antimicrobials used in human medicine. Use of PIs in rearing of production animals provides a selection pressure that promotes expansion of a PI-resistant population of bacteria and persistence of co-localized resistance mechanisms. PI-resistant bacteria can colonize humans and cause invasive infections and the PI resistance plasmids can spread in bacterial populations, both *in vitro* and *in vivo*. Therefore, there is a potential risk associated with the use of in-feed PIs, though more research is needed to explore this further to be able to conduct a thorough risk assessment with a One Health perspective.

## Author contributions

RF: Conceptualization, Data curation, Formal analysis, Investigation, Methodology, Visualization, Writing – original draft, Writing – review & editing. JS: Writing – review & editing, Conceptualization. SG: Writing – review & editing, Conceptualization. KL: Supervision, Writing – review & editing, Conceptualization. MP: Conceptualization, Data curation, Funding acquisition, Investigation, Methodology, Project administration, Writing – review & editing. AU: Conceptualization, Data curation, Formal analysis, Funding acquisition, Investigation, Methodology, Project administration, Supervision, Visualization, Writing – original draft, Writing – review & editing. RS: Conceptualization, Data curation, Formal analysis, Funding acquisition, Investigation, Methodology, Project administration, Supervision, Visualization, Writing – original draft, Writing – review & editing.
